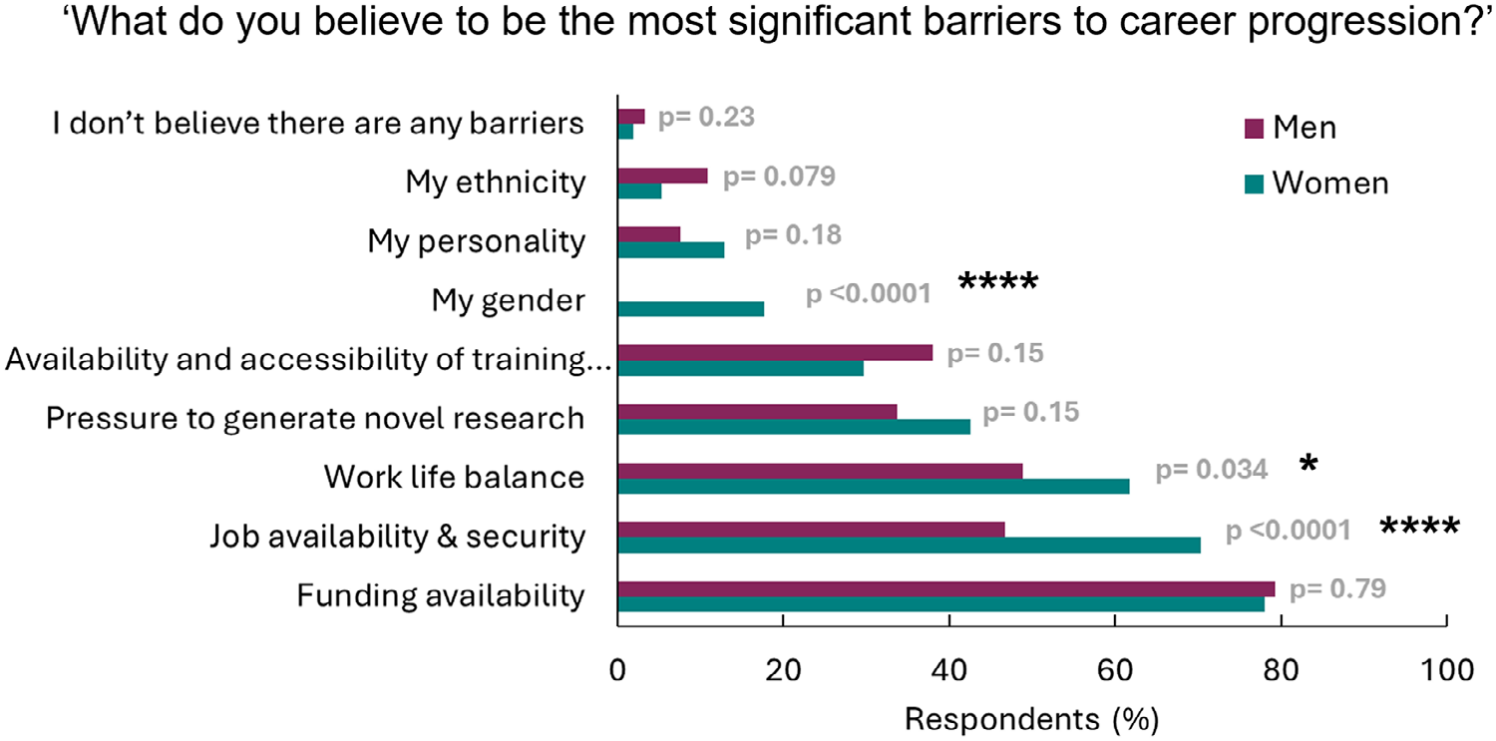# *Girls just wanna have funds and job stability in dementia research*: A cross‐sectional worldwide survey exploring gender differences in career motivations, satisfaction, perceived challenges and retention

**DOI:** 10.1002/alz70858_106659

**Published:** 2025-12-26

**Authors:** Elizabeth A. English, Sindhuja Tirumalai Govindarajan, Joshua Babalola, Sara Laureen Bartels, Nathan M D'Cunha, Shloka Dhareshwar, Charlèss Dupont, C. Elizabeth Shaaban

**Affiliations:** ^1^ University of Cambridge, Cambridge, United Kingdom; ^2^ Women In Neuroscience UK Ltd, Mildenhall, Suffolk, United Kingdom; ^3^ UK Dementia Research Institute, Cambridge, United Kingdom; ^4^ University of Pennsylvania, Philadelphia, PA, USA; ^5^ University of Texas Health Science Center, Houston, TX, USA; ^6^ Maastricht University, Maastricht, Limburg, Netherlands; ^7^ University of Canberra, Bruce, ACT, Australia; ^8^ Cardiff University, Cardiff, Wales, United Kingdom; ^9^ Redenlab. Inc, Melbourne, VIC, Australia; ^10^ End‐of‐Life Care Research Group, Vrije Universiteit Brussel (VUB) & Ghent University, Brussels, Belgium; ^11^ University of Pittsburgh, Pittsburgh, PA, USA; ^12^ University of Pittsburgh Alzheimer's Disease Research Center (ADRC), Pittsburgh, PA, USA

## Abstract

**Background:**

As a leading cause of death worldwide, research into dementia‐related diseases is crucial. Women represent two‐thirds of people with dementia, yet women are underrepresented in dementia research leadership. To explore this, our gender‐stratified study of dementia researchers investigated their motivations, role satisfaction, and perceived barriers to career progression and retention.

**Method:**

Our global survey targeted early career dementia researchers (ECDRs) through social media and email lists. Multi‐choice questions were used, often with an ‘Other’ write‐in option. Descriptive statistics were compared using chi‐square or Fisher's exact tests as appropriate. For gender comparisons, women and men were included, given the low sample size of other genders.

**Result:**

Three‐hundred‐and‐nine respondents included undergraduates to full professors: 68% women, 30% men, and 1% genderqueer, non‐binary, or self‐described. Half of respondents were considering leaving dementia research, particularly women (57% vs. 46% of men, *p* = 0.07) and ECDRs (40% of undergraduates, 61% postgraduates, 63% postdocs, 30% assistant professors, 21% full professors, *p* = 0.009). One in five women, but no men, reported their gender as one of the most significant barriers to their career progression. Job availability/security and work‐life balance were also considered career barriers by more women than men (70% vs. 47%, *p* <0.001; 62% vs. 49%, *p* = 0.034). Similarly, more women than men selected job security as one of the biggest challenges for ECDRs to stay in dementia research (90% vs. 79%, *p* = 0.04), whilst men more frequently cited the need for international experience (47% vs. 30%, *p* = 0.02). Eight female respondents had left academic dementia research, commonly because they could not find a job or funding. To return, they stated they would need longer contracts, funding opportunities and higher salaries.

**Conclusion:**

The high proportion of ECDRs considering leaving threatens dementia research progress. Women are more likely than men to consider leaving; with more female than male dementia researchers claiming their career progress and retention is disadvantaged by insufficient job availability, security, funding and work‐life balance. Targeted efforts to lessen these barriers will be essential for equitable representation and enhanced dementia research outcomes.